# Specific to Whose Body? Perspective-Taking and the Spatial Mapping of Valence

**DOI:** 10.3389/fpsyg.2013.00266

**Published:** 2013-05-13

**Authors:** Jonathan F. Kominsky, Daniel Casasanto

**Affiliations:** ^1^Department of Psychology, Yale UniversityNew Haven, CT, USA; ^2^Department of Psychology, The New School for Social ResearchNew York, NY, USA; ^3^Neurobiology of Language Department, MPI for PsycholinguisticsNijmegen, NL, Netherlands; ^4^Donders Center for Cognition, Brain, and Behavior, Radboud UniversityNijmegen, NL, Netherlands

**Keywords:** body-specificity hypothesis, handedness, perspective taking, space, valence

## Abstract

People tend to associate the abstract concepts of “good” and “bad” with their fluent and disfluent sides of space, as determined by their natural handedness or by experimental manipulation (Casasanto, 2011). Here we investigated influences of spatial perspective taking on the spatialization of “good” and “bad.” In the first experiment, participants indicated where a schematically drawn cartoon character would locate “good” and “bad” stimuli. Right-handers tended to assign “good” to the right and “bad” to the left side of egocentric space when the character shared their spatial perspective, but when the character was rotated 180° this spatial mapping was reversed: good was assigned to the character’s right side, not the participant’s. The tendency to spatialize valence from the character’s perspective was stronger in the second experiment, when participants were shown a full-featured photograph of the character. In a third experiment, most participants not only spatialized “good” and “bad” from the character’s perspective, they also based their judgments on a salient attribute of the character’s body (an injured hand) rather than their own body. Taking another’s spatial perspective encourages people to compute space-valence mappings using an allocentric frame of reference, based on the fluency with which the other person could perform motor actions with their right or left hand. When people reason from their own spatial perspective, their judgments depend, in part, on the specifics of their bodies; when people reason from someone else’s perspective, their judgments may depend on the specifics of *the other person’s body*, instead.

## Introduction

Across many cultures, the right side is associated with things that are good and lawful, and the left side with things that are dirty, bad, or prohibited. The association of “good” with “right” and “bad” with “left” is evident in positive and negative idioms like “my right-hand man” and “two left feet,” and in the meanings of English words derived from the Latin for “right” (dexter) and “left” (sinister).

Beyond patterns in language, people also implicitly associate positively and negatively valenced ideas with “right” and “left” – but not always in the way that linguistic and cultural conventions suggest. Rather, associations between valence and left-right space depend on the way people use their hands (Casasanto, 2009, 2011). When asked to decide which of two products to buy, which of two job applicants to hire, or which of two alien creatures looks more honest, intelligent, or attractive, right- and left-handers tend to respond differently: right-handers tend to prefer the product, person, or creature presented on their right side, but left-handers tend to prefer the one on their left (Casasanto, 2009). This pattern persists even when people make judgments orally, without using their hands to respond. Children as young as 5 years old already make evaluations according to handedness and spatial location, judging animals shown on their dominant side to be nicer and smarter than animals on their non-dominant side (Casasanto and Henetz, 2012).

The implicit association between valence and left-right space influences people’s memory and their motor responses, as well as their judgments. In one experiment, participants were shown the locations of fictitious positive and negative events on a map, and asked to recall the locations later. Memory errors were predicted by the valence of the event and the handedness of the participant: right-handers were biased to locate positive events too far to the right and negative events too far to the left on the map, whereas left-handers showed the opposite biases (Brunyé et al., 2012). In reaction time tasks, right- and left-handers were faster to classify words as positive when responding by pressing a button with their dominant hand, and faster to classify words as negative when responding with their non-dominant hand (de la Vega et al., 2012).

Associations of handedness with valence and space have been observed beyond the laboratory, in the speech and gestures of right- and left-handed US presidential candidates during televised debates (Casasanto and Jasmin, 2010). In right-handers, right-hand gestures were more strongly associated with positive-valence speech than left-hand gestures, and left-hand gestures were more strongly associated with negative-valence speech than right-hand gestures; the opposite associations between hand and valence were found in left-handers, despite the centuries-old tradition of training public speakers to gesture with the right hand for good things and the left hand for bad things (or not to use the left hand at all; Quintilianus, 1920).

Together, these data from studies using questionnaires, reaction time tasks, map tasks, and spontaneous gestures suggest that the association of positivity and negativity with people’s dominant and non-dominant sides of space are habitually activated, with a high degree of automaticity, when people evaluate the positivity of stimuli or recall information with a positive or negative valence. These findings provide one line of support for Casasanto’s (2009, 2011) *body-specificity hypothesis*: if the content of the mind depends, in part, on the way we interact with the environment with our bodies, then people with different kinds of bodies should tend to think differently, in predictable ways.

The body-specific association of valence with left-right space is robust, but it is also flexible. Casasanto (2009) proposed that people come to associate “positive” with their dominant side of space because they can usually interact with their physical environment more fluently on this side, using their dominant hand. This proposal follows from the finding that fluent perceptuo-motor interactions with the environment generally lead to more positive feelings, whereas disfluent interactions lead to more negative feelings and evaluations (e.g., Reber et al., 1998; Beilock and Holt, 2007; Oppenheimer, 2008; Ping et al., 2009). To test whether manual motor fluency drives associations between valence and space, Casasanto and Chrysikou (2011) studied how people think about “good” and “bad” after their dominant hand had been impaired, reversing the usual asymmetry in motor fluency between their right and left hands. This reversal of motor fluency resulted in a reversal of behavioral responses: right-handers whose right hand was impaired permanently by a unilateral stroke, or temporarily by wearing a cumbersome glove on the right hand in the laboratory, tended to associate “good” with the *left* side of space, like natural left-handers.

The finding that even a few minutes of experiencing a reversed motor asymmetry can completely reverse people’s usual judgments about the spatial mapping of valence has several implications. First, it shows that motor experience is sufficient to cause people to associate “good” with one side of space or the other, at least temporarily. Second, this finding supports a proposal at the heart of body-specificity: context shapes thinking, and the body is an ever-present part of the context in which we use our minds. To the extent that the body provides a stable context, the body-specific representations that people form are likely to appear stable over time; to the extent that body-relevant aspects of the context change, representations they activate may change accordingly (Casasanto, 2011).

To date, the body-specificity hypothesis has been tested with participants in isolating contexts: People’s brains and behaviors have been measured while they were interacting primarily with a piece of paper (Casasanto, 2009; Casasanto and Chrysikou, 2011; Casasanto and Henetz, 2012) or a computer screen (Willems et al., 2009, 2010; Brunyé et al., 2012; de la Vega et al., 2012), or while making monologic statements into a television camera (Casasanto and Jasmin, 2010). Perhaps as a consequence, the data suggest that in all of these previous studies people’s body-specific neural and mental representations have been computed from an egocentric perspective. That is, at least by default, people tend to imagine actions (Willems et al., 2009) and understand the meanings of action verbs (Willems et al., 2010) based on the way they would perform these actions with their own bodies, and they tend to activate associations between space and valence based on the long- or short-term constraints of their own manual motor fluency, using an egocentric spatial frame of reference (Casasanto, 2011).

Yet, in the richer physical and social world outside of the lab or the television studio, *other people* often feature prominently in the contexts in which we use our minds, and people often adopt other people’s mental or spatial perspectives. When communicating spatial information to another person, people frequently describe things from the recipient’s spatial perspective rather than their own (Schober, 1993, 1995; Mainwaring et al., 2003). Of particular relevance, people may spontaneously take the spatial perspective of another person depicted in a photograph when reasoning about “right” and “left,” especially when action is implied (Tversky and Hard, 2009). In face-to-face interactions, listeners tend to mimic the speaker’s bodily movements mirror-wise: if the speaker leans to her right, listeners lean to their *left*, so as to move in the same absolute direction as the speaker (but the opposite direction in body-centered space), suggesting that they spontaneously adopt an allocentric spatial perspective (Bavelas et al., 1988).

The present study investigates the consequences of spatial perspective-taking on the body-specific spatial mapping of “good” and “bad.” Although initial tests of the body-specificity hypothesis have focused on the role of one’s own body in shaping thoughts, feelings, and judgments, the idea that all thinking occurs from an egocentric perspective is ruled out by the studies reviewed above. Casasanto et al. have suggested that people may sometimes represent other people’s actions allocentrically, in terms of the specifics of *their* bodies, which are either observed or assumed (e.g., see Willems et al., 2010, p. 73; Beveridge et al., 2012). There is no doubt that people can change spatial perspectives flexibly. Here we investigated how perspective-taking interacts with the bodily characteristics of the participant and of a depicted “other” to determine judgments about the left-right mapping of “good” and “bad.”

Do people only compute space-valence mappings on the basis of their own bodily characteristics, or can they also compute these mappings on the basis of another person’s bodily characteristics (observed or assumed), when asked to reason about the other person’s choices? To find out, in three experiments, we asked right-handed participants to perform a simple diagram task, adapted from Casasanto (2009). Participants saw a character named Bob in the center of a screen in between two boxes, one on the participant’s left and the other on their right. They were asked to indicate which box Bob would put “good” things in, and which box he would put “bad” things in. For half of the participants, Bob was facing the same direction that they were (Shared Perspective condition: Bob’s right was the participant’s right), and for the other half Bob was facing the opposite direction (Opposite Perspective condition: Bob’s right was the participant’s left). All participants were instructed to reason about Bob’s placement of good and bad things *taking Bob’s perspective*.

In previous experiments, Bob was always facing the same direction as the participants. Results showed a strong tendency for left-handers to say that Bob would place good things on their left, and for right-handers to say that he would place good things on their right. This basic result in the “Bob” task has been replicated across seven experiments, conducted on three continents (Casasanto, 2009; Casasanto and Chrysikou, 2011; de la Fuente et al., 2011). We therefore expected that in the Shared Perspective condition, right-handers would tend to assign good things to the box on their right.

For the Opposite Perspective condition, we sought to distinguish two possibilities. First, participants could *still* tend to assign good things to the box on their right side. This would suggest that people’s judgments about the spatial mapping of valence are entirely egocentric: regardless of Bob’s spatial perspective (and of the explicit instructions to consider it), participants’ own motor fluency determines their responses. Alternatively, right-handed participants could tend to assign good things to the box on their left. This would suggest that participants are adopting an allocentric perspective, and reasoning about Bob’s preferences on the basis of *his* motor capacities – on the assumption (perhaps implicit) that Bob is a right-hander, which is true of about 90% of the population (Coren, 1992).

## Experiment 1: Putting Body-Specific Space-Valence Mappings in Perspective

Experiment 1 provided an initial test of the effect of spatial perspective on the left-right mapping of emotional valence, first using a simple cartoon character as in previous “Bob” experiments (Experiment 1a), and second using a more naturalistic color photograph of “Bob,” to facilitate perspective-taking (Experiment 1b).

### Experiment 1a

#### Methods

##### Participants

Three hundred adults (over 18 years old by self report) were recruited anonymously via Amazon Mechanical Turk, and participated online, for payment.

##### Materials and procedure

Materials and procedure were adapted from Casasanto (2009, Experiment 3). After providing informed consent, participants performed a two-question diagram task that has been shown to elicit contrasting space-valence judgments in right- and left-handers (Casasanto, 2009; Casasanto and Chrysikou, 2011; de la Fuente et al., 2011). Participants saw a cartoon character’s head in the center of the screen between two empty boxes, one on the participants’ right and the other on their left. They were told that the character, named Bob, loves zebras and thinks they are good, but hates pandas and thinks they are bad (or vice versa, with the assignment of valence to the animals counterbalanced across participants). Participants were asked to indicate where Bob would put each of the animals if he were going to put the good animal in one box and the bad animal in the other, by clicking inside one box and then the other. The order in which participants were asked to locate the good and bad animals was counterbalanced, to ensure that any associations between space and valence were not confounded with numerical or temporal order.

**Figure 1:**
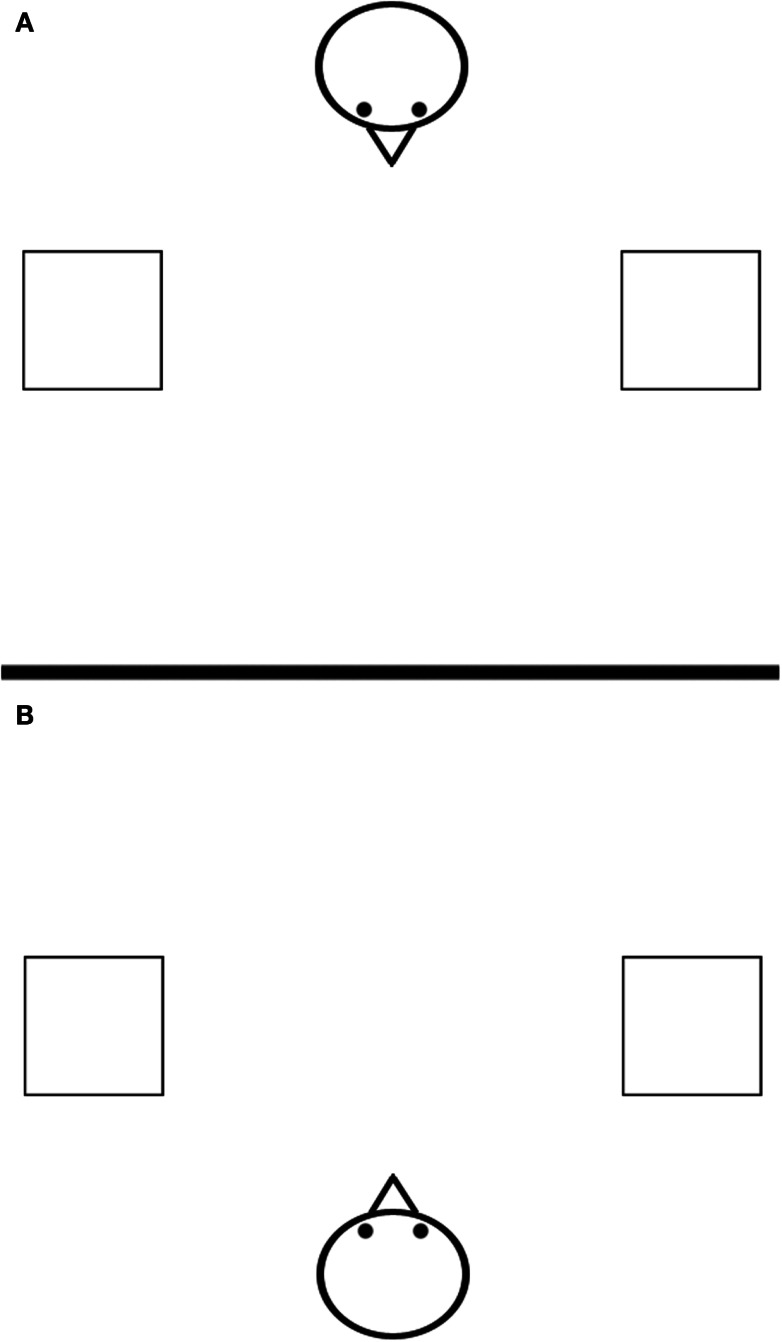
**Stimuli used in Experiment 1a for the Opposite Perspective condition [(A), top] and the Shared Perspective condition [(B), bottom]**.

Participants were randomly assigned to one of two versions of the experiment. For half of the participants, Bob was facing the same direction that they were (Shared Perspective condition; Figure 1B), and for the other half Bob was facing the opposite direction (Opposite Perspective condition; Figure 1A). All participants were instructed to take Bob’s perspective when reasoning about his placement of the good and bad animals, as in previous written versions of the “Bob” experiment (Casasanto, 2009, Experiments 1–2).

After completing the diagram task, participants answered two filler questions, and then provided a brief rationale for where they thought Bob would place the “good” animal. They then completed the Edinburgh Handedness Inventory (EHI) (Oldfield, 1971), with one added item: “Which hand do you use a computer mouse with?” This question was not used in calculating the EHI score, and was included for exploratory purposes to inform future studies. Finally, to determine whether participants were capable of taking Bob’s perspective accurately, we showed them Figure 1A and asked them to click on the box on the right (or left, randomly determined), from Bob’s point of view. Participants were then given an optional demographic questionnaire.

##### Design

The design of the experiment included three factors of interest [Valence (Good animal, Bad animal), Space (Left box, Right box), and Perspective (Shared perspective, Opposite perspective)], as well as two factors not of interest [Animal assignment (Panda = Good, Zebra = Good) and Question order (Positive animal first, Negative animal first)], resulting in a 2 × 2 × 2 × 2 × 2 design. Ideally, the design would also include a fourth factor of interest: the handedness of the participant, which would add (in the simplest case) another binary factor. However, given the rate of left-handers in the population, about 10%, we estimated that we would need a sample size of at least 1,000 participants in order to have a sufficient number of left-handers randomly assigned to each cell of the design. Fortunately, the design allows the effect of perspective-taking to be evaluated within a single handedness group, so rather than collecting a much larger sample, we decided to exclude data from all non-right-handed participants (EHI < 40).

#### Results and discussion

Left-handers (*n* = 23) and ambidextrous participants (*n* = 61) were excluded, leaving only right-handed participants (*n* = 209). Among right-handers, 91% of participants correctly answered the perspective-taking manipulation check. Of these 191 participants, 15 did not click inside of either box on the test items, so their data could not be analyzed. This left 176 participants whose data were analyzed: 85 participants in the Opposite Perspective condition and 91 in the Shared Perspective condition.

**Figure 2:**
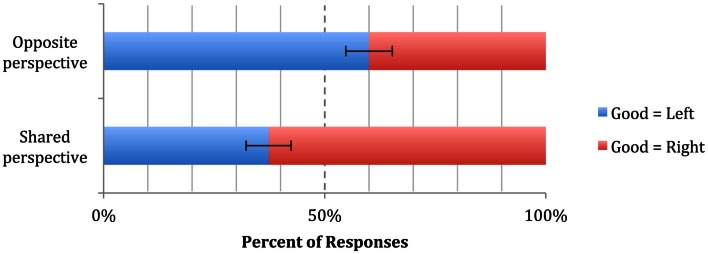
**Results of Experiment 1a**. The dashed line at 50% represents chance responding. “Good = Left” and “Good = Right” are coded from the participant’s perspective. Error bars indicate SEM.

Throughout these results, we will refer to the placement of the “good” animal from the *participant’s* perspective (i.e., egocentric right and left). In the Shared Perspective condition, the majority (63%) of participants placed the “good” animal on their right (57 right = good vs. 34 left = good, sign test *p* = 0.02), replicating previous findings in right-handers. By contrast, in the Opposite Perspective condition, the pattern was reversed, though only marginally significant, with the majority (60%) of participants placing the “good” animal on their left (i.e., on Bob’s right: 51 left = good vs. 34 right = good, sign test *p* = 0.08). A binary logistic regression confirmed the significant effect of Perspective condition on placement of the “good” animal (Wald χ^2^ = 8.86, df = 1, *p* = 0.003, OR = 2.52, 95% CI = 1.37 – 4.61), indicating that participants in the Opposite Perspective condition were about 2.5 times more likely to place the “good” animal on Bob’s right than participants in the Shared Perspective condition (Figure 2).

Analyses of the debriefing data showed that, of the participants included in the main analysis, 25% justified their assignment of the “good” animal to the right or left box on the basis of either their own handedness or Bob’s handedness. This rate was surprisingly high: in previous versions of this task, the percent of participants who explained their responses in terms of handedness has ranged from 5 (de la Fuente et al., 2011, Experiment 2) to 14% (Casasanto, 2009, Experiment 2). We do not know why the rate of debriefing responses mentioning handedness was higher in this study than in previous studies that used different versions of the same task. One possibility is that our Amazon Mechanical Turk participants, who were completing the study at their leisure, took more time to reflect on possible explanations for their choices than participants in previous studies, who were tested in the laboratory or in face-to-face conversations with the experimenter, and whose most frequent debriefing response in some studies was “I don’t know.” On this account, the increased mentions of handedness during the debriefing may not indicate that a greater proportion of participants were conscious of making their choices on the basis of handedness *during the task*; rather, these debriefing data could indicate that a greater proportion of participants generated a handedness-related explanation *post hoc*, given sufficient time to reflect on their responses. On another possibility, some of the participants in the present study may have been familiar with the idea of handedness-based space-valence associations which, since they were first reported in 2009, have been described several times in high-circulation newspapers and magazines. Whatever the correct explanation may be, we note that similar patterns of responses were found in participants who mentioned handedness during the debriefing as in those who did not. When Debriefing Response (Mentioned handedness, Did not mention handedness) was added to the binary logistic regression model, it did not interact with Perspective to predict the side of participants’ “good animal” responses (Wald χ^2^ = 2.04, df = 1, *p* = 0.15), and the effect of Perspective was still significant when the interaction of Perspective and Debriefing Response was controlled (Wald χ^2^ = 4.51, *p* = 0.03, OR = 2.02, 95% CI = 1.06 – 3.86).

In summary, when right-handed participants shared their visuo-spatial perspective with Bob, they tended to indicate that Bob would place the “good” animal on their (mutual) right side. By contrast, when Bob was rotated 180° such that his perspective was opposite the participants’, they tended to indicate that Bob would place the good animal on *his right*, which was their own left side. It appears that the association of “good” with the right side is not restricted to the egocentric right; rather, when asked to consider another’ person’s perspective, right-handers will apply the same “good is right” mapping for someone else’s point of view.

Overall, the effect of perspective on participants’ judgments was highly significant, but we note that the effect in the condition of greatest interest (Opposite Perspective) was only marginally significant, perhaps because people are not accustomed to computing the spatial perspective of a schematic, disembodied cartoon head, viewed from above. In Experiment 1b, we repeated this experiment using a full-featured photograph of “Bob,” viewed from either the back (Shared Perspective) or the front (Opposite Perspective), reasoning that the richer, more naturalistic stimulus could enhance the perspective-taking effect found in Experiment 1a.

### Experiment 1b

#### Methods

##### Participants

Three hundred new participants from Amazon Mechanical Turk participated, for payment.

##### Design, materials, and procedure

**Figure 3:**
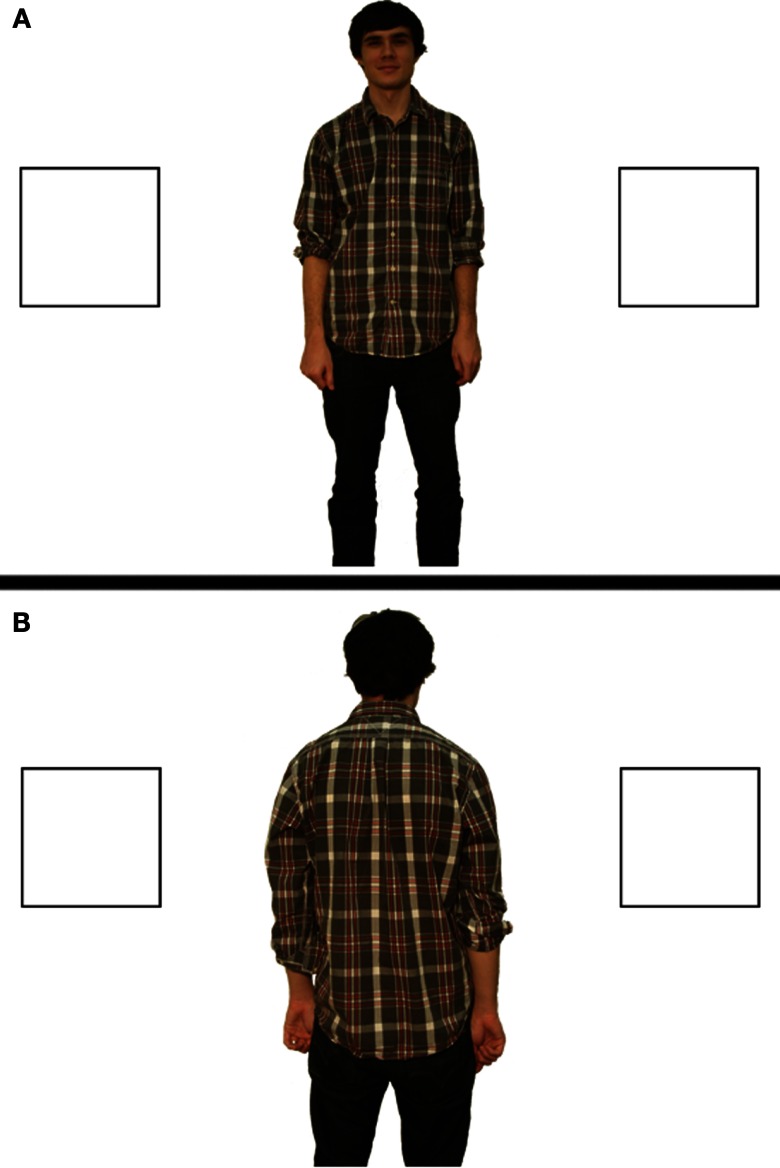
**Stimuli used in Experiment 1b for the Opposite Perspective condition [(A), top] and the Shared Perspective condition [(B), bottom]**.

The design, materials, and procedure were identical to Experiment 1a with the following exceptions: each participant saw one of the images in Figure 3, depicting a full-featured “Bob” rather than an abstract line drawing.

#### Results and discussion

After removing left-handed (*n* = 13) and ambidextrous participants (*n* = 59) there were 222 right-handed participants, 214 of whom (96%) passed the perspective-taking manipulation check. Of these participants, 6 failed to click inside the boxes, leaving 208 right-handed participants whose data could be analyzed: 112 in the Shared Perspective condition and 96 in the Opposite Perspective condition.

**Figure 4:**
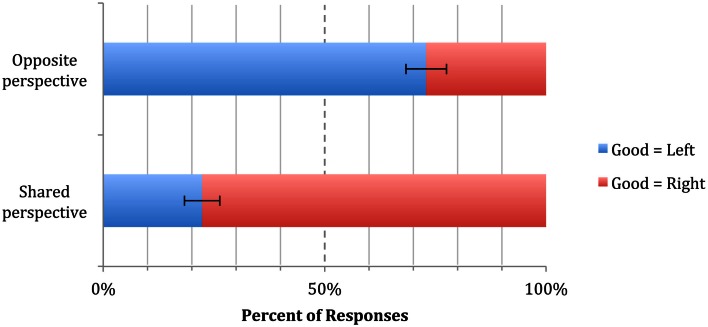
**Results of Experiment 1b**. The dashed line at 50% represents chance responding. “Good = Left” and “Good = Right” are coded from the participant’s perspective. Error bars indicate SEM.

In the Shared Perspective condition, the majority (78%) of participants indicated that the “good” animal should be placed in the box on their right (87 good = right vs. 25 good = left, sign test *p* = 0.001). In the Opposite Perspective condition, the majority (73%) of participants indicated that the “good” animal should be placed in the box on their left (Bob’s right) (70 good = left vs. 26 good = right, sign test *p* = 0.001). A binary logistic regression confirmed the effect of Perspective condition on the placement of the “good” animal (Wald χ^2^ = 48.02, df = 1, *p* = 0.001, OR = 9.37, 95% CI = 4.98 – 17.64) indicating that participants in the Opposite Perspective condition were almost 10 times more likely to place the “good” animal on Bob’s right than participants in the Shared Perspective condition (Figure 4).

Analyses of the debriefing data showed that, of the participants included in the main analysis, 42% justified their assignment of the “good” animal to the right or left box on the basis of either their own handedness or Bob’s handedness. In a further analysis, Debriefing Response (Mentioned handedness, Did not mention handedness) was added to the binary logistic regression model. There was a significant interaction between Debriefing Response and Perspective (Wald χ^2^ = 14.64, df = 1, *p* = 0.001, OR = 21.82, 95% CI = 4.50 – 105.83), indicating that the effect of Perspective was stronger in participants who explicitly mentioned handedness (Wald χ^2^ = 37.14, df = 1, *p* = 0.001, OR = 75.20, 95% CI = 18.74 – 301.74) than in those who did not, but the effect of Perspective remained significant in the majority of participants who did not mention handedness (Wald χ^2^ = 10.45, *p* = 0.001, OR = 3.45, 95% CI = 1.63 – 7.30). Pairwise differences between the number of “Good = Left” and “Good = Right” responses were significant in both the Shared and Opposite Perspective conditions, regardless of whether participants mentioned handedness in the debriefing (Table 1).

**Table 1:** **Judgments from participants who did and did not mention handedness when justifying their responses in Experiment 1b**.

Cited handedness	Perspective condition	Good = Left	Good = Right	Sign test
Yes (*n* = 88)	Shared	4	47	*p* = 0.001
	Opposite	32	5	*p* = 0.001
No (*n* = 120)	Shared	21	40	*p* = 0.02
	Opposite	38	21	*p* = 0.04

*“Good = Left” and “Good = Right” are coded from the participant’s perspective*.

The results of Experiment 1b corroborate those of Experiment 1a: when right-handers share Bob’s spatial perspective, they tend to assign the “good” animal to the box on their right and the “bad” animal to the box on their left. By contrast, when asked to decide where a 180°-rotated Bob would place the animals, participants tend to assign the “good” animal to the box on *their* *left* and the “bad” animal to the box on *their* *right*.

In order to compare the strength of the effect of Perspective between Experiments 1a and 1b, we conducted an additional binary logistic regression adding Experiment to the model used in the main analysis. The interaction of Perspective (Shared, Opposite) and Experiment (1a, 1b) was significant (Wald χ^2^ = 8.64, df = 1, *p* = 0.003, OR = 3.73, 95% CI = 1.55 – 8.96), indicating that the effect of perspective-taking effect on the spatialization of valence was stronger in Experiment 1b than Experiment 1a, presumably because participants were able to compute space-valence relationships more easily or more automatically when shown a more lifelike depiction of Bob.

As in previous tests of body-specific space-valence associations, here participants’ judgments appear to follow the “dominant side is good” mapping (whether they activate this association consciously or unconsciously). On the simplest interpretation of these data, when Bob shares their point of view, participants compute “left” and “right” from an egocentric spatial perspective, and when Bob has the opposite point of view, participants compute “left” and “right” from an allocentric spatial perspective.

Yet, there is an alternative to this conclusion. The data from the Opposite Perspective condition are consistent with participants computing “left” and “right” allocentrically, from Bob’s 180′-rotated viewpoint, *based on Bob’s bodily characteristics* – assuming (perhaps implicitly) that Bob is a right-hander, which is true of about 90% of the population (Coren, 1992). But the data are also consistent with the possibility that participants are not really considering Bob’s bodily characteristics, at all, and are instead adopting what we will call a “rotated egocentric” perspective: maybe participants are projecting their own bodily characteristics onto Bob (perhaps because they cast themselves in the “role” of Bob). In which case, in the Opposite Perspective condition they would assign the “good” animal to the box on their left, not because they assume that Bob is a right-hander (based on the handedness statistics of the population), but rather because they themselves are right-handed, and they compute space-valence associations *based on their own bodily characteristics* even when asked to reason from another person’s perspective.

Adopting a “rotated egocentric” perspective would be consistent with other demonstrations of surprising egocentrism in adults, in which experimental participants project their own bodily characteristics onto another person. For example, in one set of experiments, when asked to recall the eye color of well-known celebrities, brown-eyed participants were biased to attribute brown-eyedness to most of the stars tested, but blue-eyed participants were biased to attribute blue-eyedness to the stars, despite the rarity of blue-eyedness in the population (Casasanto and Staum Casasanto, 2011). This effect persisted when analyses were controlled for how well participants knew the celebrities, how well they liked them, and how confident participants were in their judgments: participants still tended to project their own bodily characteristics onto other people. If such egocentric projection of one’s own bodily traits onto others accounts for the results of the Opposite Perspective condition here, it would be inappropriate to conclude that switching points of view caused participants to spatialize valence from an allocentric perspective, based on Bob’s (assumed) bodily characteristics.

One way to distinguish between the “allocentric” and “rotated egocentric” possibilities would be to repeat Experiment 1 in left-handers. If participants reason about Bob’s choices from an allocentric perspective, then right- and left-handers should respond similarly in the Opposite Perspective condition, since both groups should assume that Bob is a right-hander, based on the statistics of the population. Alternatively, if participants reason from a rotated egocentric perspective, then right- and left-handers should show opposite patterns of responses in the Opposite Perspective condition: right-handers should impute right-handedness to Bob and choose the box on their left, but left-handers should impute left-handedness to Bob and choose the box on their right. Yet, there are practical and theoretical limitations to this proposed test. Practically speaking, a very large sample would be needed in order to recruit a sufficient number of left-handers from the general population. Theoretically, these imagined data would still be correlational, and therefore subject to speculations about other unexamined differences between right- and left-handers’ judgments.

In order to distinguish between the “allocentric” and “rotated egocentric” possibilities while addressing both of these concerns, for Experiment 2 we conducted a true experimental manipulation in right-handers, randomly assigning them to make judgments about Bob’s preference when provided with a highly salient indicator of his manual motor fluency with his right vs. left hand.

## Experiment 2: Are Participant’s Reasoning on the Basis of Bob’s Body or Their Own?

**Figure 5:**
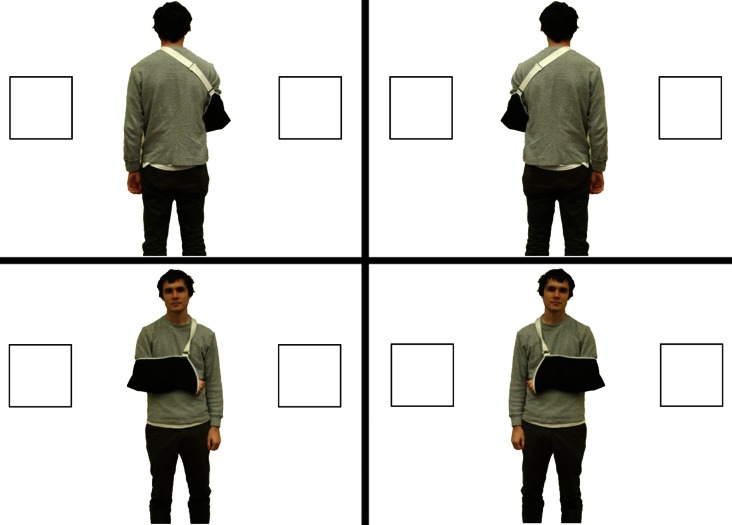
**Stimuli used in Experiment 2**. Each participant saw only one of these four images.

In order to determine whether participants in Experiment 1 were making judgments based on Bob’s bodily characteristics or their own, in Experiment 2 we asked right-handers to judge where Bob would place the good and bad animals while viewing a picture of him that made it easy to tell whether he could act more fluently with his right or left hand. Bob (viewed from either the front or the back) wore a sling on either his right or left arm, indicating that either his left hand was temporarily impaired (making him functionally a right-hander) or his right hand was impaired (making him functionally a left-hander; see Casasanto and Chrysikou, 2011; Figure 5).

If participants can reason about Bob’s spatialization of “good” and “bad” from a genuinely allocentric perspective, on the basis of Bob’s bodily characteristics, then in both the Shared Perspective and the Opposite Perspective conditions participants assigned to see Bob as functionally left-handed (sling on right arm) should respond differently from those assigned to see him as functionally right-handed (sling on left arm), since in all cases the sling makes it apparent which side is Bob’s “good” side (i.e., his fluent side). Alternatively, if participants reason about Bob’s choices from a rotated egocentric perspective, projecting their own bodily characteristics onto Bob, then the sling should have no effect on participants’ judgments. As in Experiment 1, in the Shared Perspective condition right-handed participants should put the good animal on their right, and in the Opposite Perspective condition they should put the good animal on their left (Bob’s right), regardless of which arm the sling appeared on.

### Methods

#### Participants

Six hundred new participants from Amazon Mechanical Turk participated, for payment.

#### Design, materials, and procedure

The design, materials, and procedure were identical to those in Experiment 1b, with the following exception: participants were randomly assigned to see one of the photographs in Figure 5, in which Bob, viewed from either the front or the back, wore a sling on either the right arm or the left.

### Results and discussion

Of the 469 right-handed participants who produced codable responses, 450 (96%) answered the perspective-taking manipulation check question correctly. According to a binary logistic regression, Perspective (Shared, Opposite), and Sling Arm (Right, Left) interacted to predict participants’ placement of the good animal in the box on their right or left (Wald χ^2^ = 113.86, df = 1, *p* = 0.0001, OR = 157.57, 95% CI = 62.21 – 399.11). Binary logistic regressions were then conducted for each Perspective condition, as well as sign tests for each condition.

**Figure 6:**
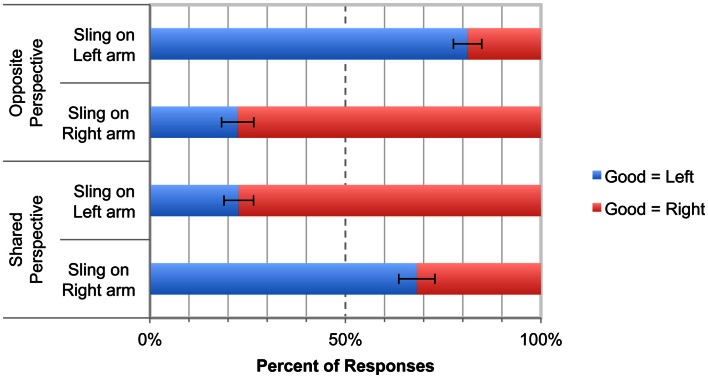
**Results of Experiment 2**. The top two bars show the results when the sling was on Bob’s left arm, and the bottom two bars show results when the sling was on Bob’s right arm. The dashed line at 50% represents chance responding. “Good = Left” and “Good = Right” are coded from the participant’s perspective. Error bars indicate SEM.

Figure 6 shows the assignment of the “good” animal in each condition. In the Opposite Perspective condition, when the sling was on Bob’s left arm, participants (*n* = 105) put the “good” animal on their left 84% of the time (88 good = left vs. 17 good = right, sign test *p* = 0.001). When the sling was on Bob’s right arm, participants (*n* = 107) put the good animal on their left only 22% of the time (24 good = left vs. 83 good = right, sign test *p* = 0.001; Wald χ^2^ = 67.17, *p* = 0.001, OR = 17.90, 95% CI = 8.98 – 35.69).

In the Shared Perspective condition, when the sling was on Bob’s left arm, participants (*n* = 112) put the “good” animal on their right 80% of the time (90 good = right vs. 22 good = left, sign test *p* = 0.001), whereas when the sling was on Bob’s right arm (*n* = 104), participants put the good animal on their right only 32% of the time (33 good = right vs. 71 good = left, sign test *p* = 0.001; Wald χ^2^ = 46.86, *p* = 0.001, OR = 8.80, 95% CI = 4.72 – 16.41).

In summary, in both the Shared Perspective and Opposite Perspective conditions, the majority of participants assigned the “good” animal to Bob’s fluent side of space, that is, the side ipsilateral to his sling-free arm. Results suggest that participants in the Opposite Perspective condition were adopting a genuine allocentric perspective, not a rotated egocentric perspective, and basing their judgments on Bob’s bodily characteristics rather than their own.

## General Discussion

In two experiments, we demonstrated that taking another person’s perspective can influence judgments about the spatial mapping of emotional valence. In Experiment 1, when participants shared the same spatial point of view as the cartoon character whose preferences they were asked to reason about, the (right-handed) participants tended to compute space-valence relationships egocentrically, showing the “good is right” bias found previously in right-handers (Casasanto, 2009). That is, participants indicated that the “good” side of space was the side on which they could interact with the physical environment more fluently using their dominant hand. When the participants’ point of view was rotated 180° from the character’s, however, the spatial mapping of valence was reversed: “Good” was assigned most often to the character’s right side (i.e., the participant’s left). This effect of spatial perspective was strengthened when the cartoon character used in Experiment 1a was replaced with a color photograph of a man (Experiment 1b), presumably because the full-featured photograph enabled participants to compute space-valence relationships from the character’s perspective more easily or more automatically.

The results of Experiment 1 were compatible with two possibilities: when participants computed space-valence relationships they could have been adopting an allocentric perspective, basing their judgments on the character’s bodily characteristics (assuming, perhaps implicitly, that the character was right-handed, like the 90% majority of people). Alternatively, they could have been adopting a “rotated egocentric” perspective, projecting their own handedness onto the character or putting themselves in his shoes, and basing their judgments on their own bodily characteristics. In Experiment 2, the character’s hand dominance could be inferred unambiguously. Results clearly indicated that participants’ were adopting an allocentric perspective: they spatialized “good” and “bad” on the basis of the character’s bodily characteristics, not their own.

Is it possible that the results of these experiments were artifacts of the particular task used, in which participants were explicitly asked to spatialize “good” and “bad,” and to use their hands when responding? For example, could clicking the mouse on one’s dominant side of the screen have been easier than clicking on the other side, leading to a trivial association between space and valence? It is unlikely that such task characteristics can explain these results, for several reasons. First, there is no reason to believe that using the mouse with one’s dominant hand should cause participants to prefer to click in a particular box, or that it was easier to click in one box vs. the other. Furthermore, even if responding with the mouse did bias people toward responding on one side of the screen (e.g., if it were slightly easier to click on one side than the other), the fact that participants’ preferred box *reversed* according to whether Bob was facing toward them or away from them definitively rules out any explanation for their responses based on the location of the mouse, or how easy it was to click in the right or left box.

More broadly, across previous studies using the “Bob goes to the zoo” task, results obtained in versions of that task that required manual responses (e.g., Casasanto, 2009, Experiments 1–2) have been statistically indistinguishable from results of “hands-free” versions that only required oral responses (e.g., Casasanto, 2009, Experiment 3; de la Fuente et al., 2011), addressing concerns about the use of the hands during this task. More broadly still, we note that the fluency-based body-specific association of left-right space and valence has been shown in a wide variety of tasks with diverse dependent measures (e.g., diagram tasks, forced-choice questionnaires, reaction time tasks, visual-hemifield tasks, location memory tasks, analyses of spontaneous gestures), and in diverse populations including healthy right- and left-handed adults from the USA, Germany, The Netherlands, Spain, and Morocco, as well as hemiparesis patients, children as young as 5 years old, and US presidential candidates who did not know that they were experimental “participants” (Casasanto, 2009; Casasanto and Jasmin, 2010; Brookshire and Casasanto, 2011; Casasanto and Chrysikou, 2011; de la Fuente et al., 2011; Brunyé et al., 2012; Casasanto and Henetz, 2012; de la Vega et al., 2012).

We acknowledge, however, that this study is only a first test of the effects of spatial perspective on left-right valence associations. It would be useful to corroborate these results with further tests that use more implicit dependent measures, which could rule out other potential task-based explanations. For example, an anonymous reviewer suggested this alternative account of the findings of Experiment 2: rather than reasoning about “good” and “bad” from Bob’s perspective, participants could have used a simple matching strategy, matching the good animal with the “good” (i.e., uninjured) side of Bob’s body. Although our data cannot rule out this possibility, such an explanation cannot account for the perspective-taking evident in Experiment 1, or the results of the several previous versions of the “Bob goes to the zoo” task reviewed above.

One question left open by this study is: to what extent do people take another’s spatial point of view spontaneously, and therefore reason about space and valence from an allocentric perspective, based on characteristics of the other person’s body? In these experiments, we explicitly instructed participants to adopt the character’s perspective (and made sure they were capable of doing so correctly). Yet, even without explicit instruction, people routinely represent the perspective of people with whom they interact face-to-face, as is evidenced by studies of dialog (e.g., Schober, 1995), gesture (McNeill, 1992), mimicry (Bavelas et al., 1988), and spatial descriptions of pictures (Mainwaring et al., 2003; Tversky and Hard, 2009). It is likely, therefore, that allocentric reasoning about space and valence may occur spontaneously.

Another open question is the extent to which people’s reasoning about space and valence is constituted by modality-specific simulations of motor actions, and the fluency with which they could be performed on one side of space or the other. Previous studies show that people imagine actions and understand decontextualized action verbs, in part, via motor simulations constructed from a body-specific, egocentric perspective. That is, when asked to imagine “grasping” or to read the verb “grasp,” right- and left-handers preferentially activate motor areas in the hemisphere contralateral to their dominant hand that are used for planning and performing manual actions (Willems et al., 2009, 2010). There is clear evidence that the body-specific spatialization of emotional valence depends on an individual’s history of motor actions (Casasanto and Chrysikou, 2011). It is not known, however, whether the mental representations that underlie reasoning about the spatial correlates of valence are constituted, in part, by motor simulations: that is, motor simulations of actions as people would perform them with their own bodies (when they adopt an egocentric perspective), and motor simulations of relatively fluent and disfluent actions as they would be performed by another (when they adopt an allocentric perspective). Determining whether the representations underlying behavioral effects like those we show here include simulations in motor circuits that support acting with the dominant and non-dominant hands will require more direct observations of neural activity (e.g., using fMRI) or direct interventions on neural circuits that compute hand actions (e.g., using rTMS or tDCS).

## Conclusion

These experiments corroborate previous studies showing that the spatial mapping of “good” and “bad” is body-specific. Furthermore, they show for the first time that the body this spatial mapping is *specific* *to* is not necessarily one’s own. When we reason from our own perspective, our judgments are conditioned by the particulars of our bodies; when we reason from someone else’s perspective, our judgments may be conditioned by the particulars of *their* bodies. The body shapes our thoughts, feelings, and judgments because it is an ever-present part of the context in which we use our minds (Casasanto, 2011). Other people are also an important element of the context in which we do our thinking, therefore thinking is sensitive to the specifics of their bodies, as well as our own.

## Conflict of Interest Statement

The authors declare that the research was conducted in the absence of any commercial or financial relationships that could be construed as a potential conflict of interest.
